# Psychotropic Drug Use in Children and Adolescents Before and During the COVID-19 Pandemic

**DOI:** 10.1001/jamapediatrics.2021.5634

**Published:** 2022-01-04

**Authors:** Christine Leong, Laurence Y. Katz, James M. Bolton, Murray W. Enns, Joseph Delaney, Qier Tan, Jitender Sareen

**Affiliations:** 1 University of Manitoba, Winnipeg, Manitoba, Canada; 2 Manitoba Centre for Health Policy, Winnipeg, Manitoba, Canada

## Abstract

This cross-sectional study examines incidence of prevalence psychotropic drug use in Manitoban children before and during the COVID-19 pandemic.

Children have generally experienced less severe physical symptoms of COVID-19 than the adult population.^1^ However, the mental health effects appear to be profound.^2,3^ It is not certain whether the COVID-19 pandemic is associated with the use of psychotropic drug medication in children. This study aimed to examine the extent of change in psychotropic use in children before and during the pandemic. It is hypothesized that psychotropic drug use would be associated with the pandemic.

## Methods

Data from the Manitoba Centre for Health Policy Repository were used for this cross-sectional study to examine the quarterly incidence and prevalence of psychotropic use in children 18 years or younger from January 1, 2015, to December 31, 2020. Medication dispensations are recorded for all Manitobans regardless of age or income. Psychotropic classes based on World Health Organization anatomic therapeutic chemical categories included antidepressant, anxiolytic/sedative-hypnotic, antipsychotic, and stimulant. Incidence was defined as no dispensation in the previous 3 years. The proportion of children receiving a drug from psychotropic drug within each quarter of 2020 was compared with the mean proportion receiving the same psychotropic class in the same quarter in the 5 years prior (2015 to 2019). This study was approved by the University of Manitoba Human Research Ethics Board and Health Information Privacy Commission to allow analysis of deidentified data and waiver of consent. Strengthening the Reporting of Observational Studies in Epidemiology (STROBE) reporting guidelines were used. Analyses used 2-tailed t tests (SAS, version 9.4; SAS Institute) to test for significant mean differences between 2020 and previous years, stratified by quarter with *P* < .05 indicating significance.

## Results

Demographics for the first quarter of 2020 are described in the Table. Incident stimulant (*P* = .001) and anxiolytic/sedative-hypnotic (*P* = .02) medications had the greatest decline in use in quarter 2 of 2020 (2.38 per 1000 individuals and 1.03 per 1000 individuals, respectively) compared with the mean incident use in quarter 2 in the previous years (4.73 per 1000 individuals [range, 4.43 per 1000 individuals to 5.12 per 1000 individuals] and 1.79 per 1000 individuals on average [range, 1.65 per 1000 individuals to 2.08 per 1000 individuals], respectively) (Figure, A). Incident antidepressant use was nonsignificantly higher in the fourth quarter of 2020 (3.61 per 1000 individuals) compared with the incidence in quarter 4 of previous years (mean, 2.93 per 1000 individuals; range, 2.57 per 1000 individuals to 3.25 per 1000 individuals) (*P* = .13). Prevalent stimulant and anxiolytic/sedative-hypnotic use showed similar patterns but was not statistically significant (*P* = .36 and *P* = .05, respectively) (Figure, B). Antidepressant prevalence was also nonsignificantly higher in quarter 4 of 2020, compared with previous years (*P* = .13) (Figure, B).

**Table.** Study Population Demographic Characteristics and Timeline of Policy Changes in Manitoba, Canada

Characteristic	Individuals, No. (%)
No.	330 398
Age, mean (SD), y	8.9 (5.4)
Age group, y	
0-5	104 719 (31.7)
6-12	125 791 (38.1)
13-18	99 888 (30.2)
Sex	
Female	160 971 (48.7)
Male	169 427 (51.3)
Urban residence	183 531 (55.9)
Income quintile	
Rural	
1 (Lowest)	34 988 (10.6)
2	29 004 (8.8)
3	25 801 (7.8)
4	28 175 (8.5)
5 (Highest)	27 768 (8.4)
Urban	
1 (Lowest)	37 194 (11.3)
2	26 637 (11.1)
3	34 108 (10.3)
4	36 242 (11.0)
5 (Highest)	37 457 (11.3)
Not found	3024 (0.9)
Psychiatry disorder diagnosis in past 5 y	61 759 (18.7)
Mood disorder/anxiety	29 485 (8.9)
Psychosis	7073 (2.1)
Schizophrenia	152 (0.1)
Personality disorder	393 (0.1)
Timeline of policy changes in Manitoba in 2020	
March 12, 2020	First COVID-19 case in Manitoba.
March 16, 2020	Closure of universities, childcare services, nonessential service operations, events consisting of >50 people prohibited
March 20, 2020	Manitoba premier declared a state of emergency for 30 d
March 30, 2020	In-person kindergarten to grade 12 classes changed to remote learning

**Figure.**
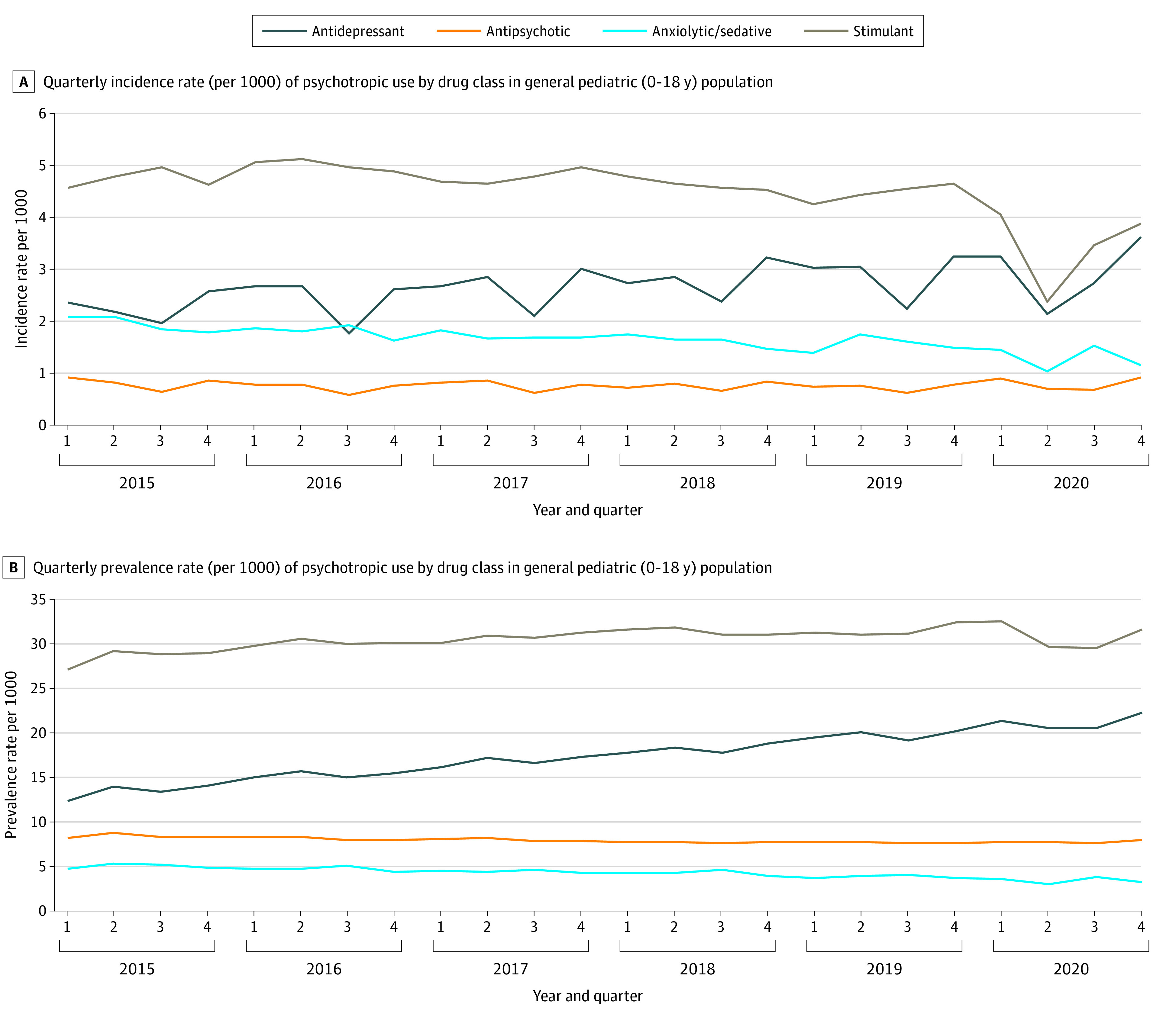
Incidence and Prevalence of Psychotropic Drug Use in the General Pediatric Population A, Quarterly incidence rate (per 1000) of psychotropic drug use by drug class in the general pediatric (age ≤18 years) population. B, Quarterly prevalence rate (per 1000) of psychotropic use by drug class in the general pediatric (age ≤18 years) population.

## Discussion

This population-based study found an almost 2-fold decline in incident stimulant and anxiolytic/sedative-hypnotic use among children in the quarter immediately after public health closures in 2020 compared with the same quarter in previous years. Peak rates are expected in quarter 1 (January to March) of each year because Manitobans receive full coverage for eligible medications after an income-based deductible is met, which resets in April. Quarter 2 rates of 2020 were significantly lower for stimulants and anxiolytic/sedative-hypnotics than in previous years. This may be expected for stimulant use as a result of school closures during this period. Moreover, in-person physician visit restrictions may have impeded the ability to diagnose and start new prescriptions during this period. Interestingly, a trending increase in antidepressant use above prepandemic rates in the last quarter of 2020 was observed. This could reflect an increase in new-onset depression or anxiety in this population during this period. Prevalence rates demonstrated statistically nonsignificant similar trends.

Strengths of this study included the ability to examine psychotropic drug use in a population-based sample of children using data not restricted by drug coverage. This study also looked back 5 years to account for the changing trend in use. Limitations include that the study team did not examine psychotropic medication indication. Further studies are needed to understand whether these changes are associated with the health of children in the long term.
